# Wetland environmental bioreactor system contributes to the decomposition of cellulose

**DOI:** 10.1002/ece3.5326

**Published:** 2019-07-02

**Authors:** Wen Liu, Aya Tanimura, Yumi Nagara, Tetsuhiro Watanabe, Shingo Maegawa, Haruhiko Toyohara

**Affiliations:** ^1^ Graduate School of Global Environmental Studies Kyoto University Kyoto Japan; ^2^ Division of Applied Biosciences, Graduate School of Agriculture Kyoto University Kyoto Japan; ^3^ Division of Environmental Science and Technology, Graduate School of Agriculture Kyoto University Kyoto Japan; ^4^ Department of Intelligence Science and Technology, Graduate School of Informatics Kyoto University Kyoto Japan

**Keywords:** cellulose breakdown, *Corbicula japonica*, environmental cellulase, invertebrate cellulase, wetland

## Abstract

Recently, numerous species of aquatic invertebrates inhabiting wetlands have been shown to possess endogenous cellulase, following the discovery that termites have cellulase genes encoded in their own genome rather than relying on symbiotic bacteria for decomposing cellulose. Wetlands have been empirically shown to play an important role in the decomposition of land‐originating hard‐to‐degrade polysaccharides such as cellulose. However, the mechanism that connects the cellulase producer and the wetlands remains unknown, which makes it very difficult to evaluate the ecological function of wetlands. Here we found that a macrobenthic bivalve, *Corbicula japonica*, secretes its cellulase to the wetland sediment. Secreted cellulases are immobilized in the components of the sediment. Moreover, adding cellulose or glucose to *C. japonica* could trigger its cellulase secretion level. These findings suggest a novel wetland cellulose decomposition mechanism. The decomposition ability of wetlands was previously ascribed only to microbes and/or invertebrates that contain cellulases. Our findings suggest that benthic animals supply wetlands with their enzymes as decomposition agents, while wetland sediments serve as immobilization scaffolds for the enzymes. This system, which was named by us an “environmental bioreactor system,” could provide a key function in wetlands.

## 1 INTRODUCTION

Cellulose, the most abundant organic matter on the earth, is a product of photosynthesis by plants and phototrophic bacteria (Watanabe & Tokuda, 2001). It is very difficult for this polysaccharide to decompose because of its stable chemical linkages inside a monomeric cellulose chain, as well as the crystalline structure formed by multiple cellulose microfibrils that are interconnected by hydrogen bonds (Tomme, Warren, & Gilkes, 1995). A special group of enzymes collectively called cellulases is required to decompose cellulose into the component monosaccharide, namely glucose. Cellulases are classified into several groups according to their functions, such as the cleavage site in cellulose (Tanimura, Liu, Yamada, Kishida, & Toyohara, 2012). Cellulases were considered to be produced only by microorganisms until the discovery of an endogenous cellulase gene that belongs to a termite, *Reticulitermes speratus* (Watanabe, Noda, Tokuda, & Lo, 1998). After that, a variety of invertebrates inhabiting wetlands were proved to possess endogenous cellulases, including organisms in the phyla Annelida, Mollusca, Arthropoda, and Echinodermata so far (Tanimura et al., 2012).

Wetlands, including coastal/tidal wetlands, are transitional ecosystems between upland and aquatic ecosystems, and have soils that are saturated and can thus support hydrophytic vegetation. Due to the intrinsic factors that define wetlands, wetlands are generally considered carbon sinks resulting in storage (i.e., high carbon density) relative to other ecosystems globally (Scholz, 2011; Sudip, Reiner, & Paul, 2005). In addition to autochthonous carbon production, wetlands also receive carbon inputs from upstream ecosystems (allochthonous C inputs) (Pant, Rechcigl, & Adjei, 2003).

In our prior unpublished experiment, it was determined that cellulase activity in the sediment was not reduced after long‐term refrigeration for more than half a year (W. Liu, unpublished experiment). Further study indicated that cellulase activity in the sediment could remain after removing all invertebrates and suppressing microbial growth by adding a protein synthesis inhibitor (chloramphenicol) as an antibiotic (Liu & Toyohara, 2012). This remaining activity, defined as “environmental cellulase activity,” could be derived in part from the intracellular cellulase from microbes that were present in the sediment prior to the addition of antibiotics. However, the cellulase of this origin is considered to be minor.

Besides microbes, invertebrates are also abundant in wetlands (Batzer, Rader, & Wissinger, 1999). Their possession of endogenous cellulase indicates their potential involvement in cellulose decomposition in wetlands. Prior studies revealed that various invertebrates are involved in leaf litter decomposition, although many of the studies used a macroecological approach, such as studies of the relationship between the macroinvertebrate abundance and leaf species (Tiegs, Entrekin, Reeves, Kuntzsch, & Merritt, 2013), or the differences of assimilation efficiency between species or leaf litter with different nutritional qualities (Stoler, Golembieski, Stephens, & Raffel, 2016). Using another approach, stable isotope analysis showed that our model invertebrate, *Corbicula japonica*, utilizes land‐originating organic matter, which in most cases is plant residues (Kasai & Nakata, 2005). Given these results, a connection between the cellulase‐possessing bivalves and the cellulose decomposition process in the wetlands was suggested, but the detailed mechanisms of exactly how these invertebrates assimilate cellulose have not been well studied and fully clarified. These facts also indicate the possibility that environmental cellulase activity could be derived from cellulase secreted by invertebrates. Based on these facts, we formed the hypothesis that some invertebrates secrete cellulase into the sediment, and the secreted cellulase somehow becomes immobilized and remains functional in the sediment, and is thus responsible for “environmental cellulase activity.” To test this hypothesis, here we attempted to detect one of *C. japonica*'s cellulases (Cjcel9A) using a specific antibody. We also cultured *C. japonica* individuals in a controlled environment to see whether they could secrete cellulase(s) in laboratory conditions. Having verified that Cjcel9A is secreted and can be immobilized in the sediment, we examined which component(s) of sediment immobilize secreted cellulase(s) and could thus prevent the cellulase being washed away. Based on the results obtained here with *C. japonica*, we finally discuss the origin of “environmental cellulase activity,” and the contribution of such activity to the function of wetlands.

## 2 MATERIALS AND METHODS

### 2.1 Materials

Sediment samples for cellulase activity detection and immunological detection, reed (*Phragmites australis*) leaves, and *C. japonica* were collected from an untouched wetland located in Tanaka River Estuary (TRE), Mie Prefecture, Japan (Kimura & Kimura, 1999). In addition to the sediment from TRE, sediments from river estuaries, ponds, lakes and land in Hokkaido, Honshu, and Okinawa Islands in Japan were also collected for the investigation of the correlation between cellulase‐binding ability and sediment texture. All samples were transported to and stored in the laboratory at 4°C until use. *Corbicula japonica* were cultured at 20°C in 50% artificial seawater (REI‐SEA Marine II, IWAKI, prepared according to the manufacturer's directions and adjusted to 3.5% salinity, same as below) and fed diatoms (*Chaetoceros constrictus*, *Chaetoceros didymus*, *Chaetoceros socialis*, and *Chaetoceros socillis* mixture) until use.

### 2.2 Cellulase activity measurement

Reducing sugar production was measured according to the tetrazolium method reported previously (Jue & Lipke, 1985). Sodium acetate buffer (0.2 M, pH 5.9) with 1% carboxymethylcellulose (CMC) (Sigma) in the presence of 10 mM NaN_3 _was used in all experiments using the cellulase activity measurement system (CAMS). One unit of cellulase activity is defined as the enzyme activity that could produce reducing sugar from CMC with the same reducing power as 1 µg of glucose in 1 min at 25°C. Glucose concentrations were measured using an EnzyChrom Glucose Assay Kit (BioAssay Systems) following the manufacturer's protocol.

### 2.3 Recombinant protein expression and purification

The carbohydrate binding module (CBM) sequence of *Cjcel9A* (GenBank: AB264777.1) was amplified using primers 5′‐GAGGGATCCC(CAC)×6GCACCAGTAACTATC‐3′ and 5′‐GAGGGTACCCCTGGACCTACAGACCT‐3′ (the underlined sequence is complementary to the *Cjcel9A* CBM), inserted into pEGFP plasmid (BD Biosciences Clontech) and transformed into the *XL‐blue* strain of *E. coli* for expression. The recombinant protein was purified using a His‐trap™ FF column (GE Healthcare) following the manufacturer's protocol.

### 2.4 Antibody purification

Purified recombinant His_6_‐CBM was used for purifying the anti‐Cjcel9A antibody produced in our previous study (Sakamoto, Uji, Kurokawa, & Toyohara, 2008). His_6_‐CBM was desalinized using a prepacked disposable PD‐10 column (GE Healthcare) before immobilization on a HiTrap NHS‐activated HP column (GE Healthcare). Antiserum was concentrated using a 5,000 Da molecular sieve (Amicon Ultra‐15 5,000 MWCO, Millipore) before purification.

### 2.5 Secreted cellulase activity

Sediments collected from four sites in TRE were sieved (45 µm mesh) to remove all invertebrates. Then, chloramphenicol was mixed with sediment to a final concentration of 40 mg/g sediment (wet weight). Cellulase activity was measured after a 24 hr incubation in a 10 ml CAMS.

### 2.6 Detection of *C. japonica* cellulase (Cjcel9A) in sediment

Surface sediment (*ϕ* = 5 cm, depth = 1 cm) surrounding 10 *C. japonica* individuals was collected. All samples were mixed, washed with 500 ml autoclaved artificial seawater (AAS), then supplemented with a detergent (sodium dodecyl sulfate, SDS) at final concentration of 0.1%, and mixed vigorously for total protein extraction. After the mixture was allowed to stand for 1 hr, the supernatant was concentrated approximately 200‐fold using a 5,000 Da molecular sieve, and then used for Western blotting. Anti‐rabbit IgG (H&L) HRP‐linked antibody (Cell Signaling Technology) was used as the secondary antibody, and high‐performance autoradiography film (Amersham^®^ Hyperfilm^®^, GE Healthcare) was used for imaging.

### 2.7 Proportion of cellulase activity present in plant residues and mineral fractions

Visible plant residues were picked out from the sediment using tweezers under an optical microscope. The cellulase activity in the collected plant residues and remaining minerals was measured, respectively.

### 2.8 Correlation between cellulase‐binding ability and soil texture of the sediments

One hundred milligrams of each sample was supplemented with 100 units of *C. japonica* cellulase. *Corbicula japonica* cellulase was prepared by collecting and dissolving crystalline styles, an enzyme storage organ, in 1 ml CAMS buffer, and mixed at 4°C for 3 hr. After the supernatant was discarded, the sediment was washed with 1 ml AAS 5 times, and the remaining cellulase activity was measured and expressed as the bound cellulase activity (units/g, dry weight).

The organic matter content of the sediments was determined as the ignition loss rate at 600°C for 3 hr. The clay contents were determined using the pipette method, and the sand content was determined using the sieving method as described in a previous report (Dane & Topp, 2002). The iron and aluminum contents were measured by the acid ammonium oxalate extraction assay and pyrophosphate extraction assay according to a previous report (Blakemore, Searle, & Daly, 1987).

### 2.9 Reconstruction experiment to reproduce the secretion and immobilization of environmental cellulase

Autoclaved reed leaves/cellulose were immersed inside a sieve (45 µm mesh, *ϕ* = 75 mm) for easy collection in a culture tank containing 2 L AAS and 25 *C. japonica* individuals. After 24 hr, leaves (1mg, 100 µl CAMS, *n* = 3) and cellulose (25 mg/duplicate, 100 µl CAMS, *n* = 5) were collected and washed with 1 ml AAS 5 times before measuring reducing sugar production or glucose production. For Western blotting and immunofluorescence, 5 mg cellulose was used for each assay. Alexa Fluor 546 (Life Technologies) was used as the secondary antibody for immunofluorescence.

### 2.10 Effect of poly‐, oligo‐, and monosaccharides on cellulase secretion

Glucose, cellobiose, cellotriose, cellotetraose, cellopentaose, cellohexaose, cellulose, CMC, xylan (birchwood), or pectin (citrus) was added to a total of 3 ml of artificial seawater containing a single *C. japonica* (*n* = 3) at the final concentration of 1, 10, 100, or 1,000 µg/ml. After 24 hr, cellulase activity in the seawater was measured.

### 2.11 Glucose uptake

*Corbicula japonica* (2, 4 or 6 individuals per group, *n* = 3) were cultured in 100 ml of artificial seawater containing 1 mM glucose and 1 mM NaN_3_. In the control group, *C. japonica* individuals were substituted by their shells after they had been sterilized with 70% ethanol. The glucose concentration in the seawater was monitored by using the tetrazolium method.

### 2.12 Estimation of the contribution of *C. japonica* to the cellulase activity in wetland sediments

The contribution of *C. japonica* to the wetland sediment in the Sandy field of TRE was approximated as follows:

(1) (ECS×N)/ACA×100%

**Figure 1:**
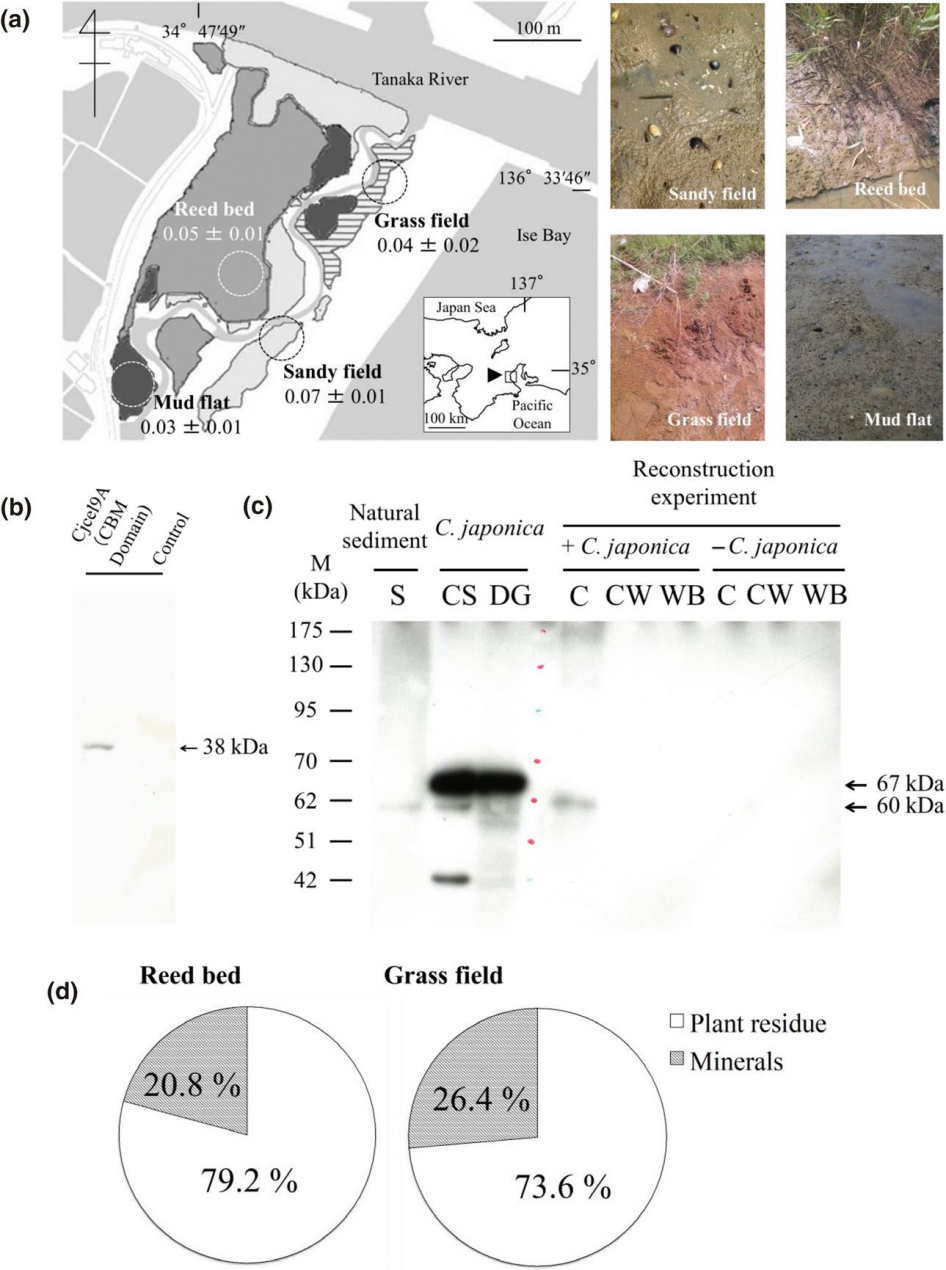
“Environmental cellulases” in wetland sediment. (a) Environmental cellulase activities in the sediment of 4 sites (Sandy field, Reed bed, Grass field, and Mud flat) were investigated by measuring the reducing sugar produced when the substrate carboxymethyl cellulose (CMC) was added after removing the macrobenthos (≧2 mm) and meiobenthos (≧0.45 mm, <2 mm) with sieves, and suppressing the microorganisms’ cellulase activities with chloramphenicol at the final concentration of 40 mg/g sediment (wet weight). The environmental cellulase activities are expressed as units/g sediment (dry weight). Values are mean ± *SEM* (*n* = 3). (b) Verification of Cjcel9A antibody. Purified Cjcel9A antibody recognizes the carbohydrate binding module (CBM) in Cjcel9A. The CBMs are specific amino acid sequences that sometimes exist beside the catalytic domains of cellulase. A CBM domain was verified to exist in the N terminus of Cjcel9A's catalytic domain in our previous study (Sakamoto, Touhata, Yamashita, Kasai, & Toyohara, 2007). We used its recombinant protein (10 µg of His_6_‐CBM) to verify the binding of the antibody. (c) Immunological detection by Western blotting of environmental Cjcel9A cellulase and secreted Cjcel9A in reconstruction experiment (7.5% acrylamide gel, primary anti‐Cjcel9A antibody diluted 1:1,000, secondary anti‐rabbit IgG HRP‐linked antibody diluted 1:10,000). S, sediment collected near the habitats of *Corbicula japonica* in TRE; CS/DG, crystalline style/digestive glands of *C. japonica* (positive control); C, cellulose collected in the reconstruction experiment shown in Figure 3; CW, culture water in the reconstruction experiment (control); WB, washing buffer of cellulose after it was collected from the reconstruction experiment (control). The 60 kDa form of Cjcel9A detected in the crystalline style (Lane CS) or digestive glands (Lane DG) of *C. japonica* was proved to exist in the natural sediment of TRE (Lane S), as well as to be secreted in the reconstruction experiment (Lane C, in the presence of *C. japonica*). The signal of Cjcel9A was detected neither in the culture water (Lane CW) nor in the washing buffer, indicating that most of the secreted Cjcel9A was bound to cellulose and difficult to wash off. Signals of 67 kDa in the crystalline style and digestive glands could be a precursor of Cjcel9A, while signals smaller than 60 kDa could be Cjcel9A hydrolysates. (d) Immobilization scaffolds of the secreted cellulases. Proportion of cellulase activity of the plant residues and remaining minerals relative to the activity of total sediment (*n* = 3, dry weight) in the sites of the Reed bed and Grass field in TRE. More than 70% of the cellulase activities were associated with the plant residues

ECS: Efficiency of cellulase secretion in one day (units per individual). *N*: Number of individuals (per m^3^). ACA: Average cellulase activity (units per cm^3^) = Average cellulase activity (units g^−3^, calculated by averaging results of the four sites shown in Figure 1, and weighted according to the proportion of their area, which was measured using ImageJ, ver.1.51a) × density of sediment.

### 2.13 Statistical analyses

Statistical analyses were carried out using IBM SPSS, ver.22. Bivariate correlation analysis (Pearson correlation coefficients, 2‐tailed) was used to examine the correlation between the cellulase‐binding ability and soil texture of the sediments. Levene's Test was used before one‐way ANOVA. Tukey's HSD post hoc analysis was applied to the results of reducing sugar production, glucose production, and glucose uptake. Scheffe post hoc analysis was applied to determine the effect on cellulase secretion.

## 3 RESULTS

### 3.1 Environmental cellulase activities and a bivalve cellulase provider

First, we found that all of the sites investigated in TRE contained environmental cellulase activities (Figure 1a). These activities were considered to be derived from microbes and/or cellulase‐possessing invertebrates. Next, we focused on the Sandy field in TRE, in which a local bivalve species, namely *C. japonica*, was dominant. By targeting the carbohydrate binding module (CBM) of one of its cellulases, namely Cjcel9A (Figure 1b), we detected the presence of Cjcel9A in the sediment of TRE immunologically by Western blotting (Figure 1c, Lane S).

### 3.2 Immobilization scaffolds of the environmental cellulases

**Figure 2:**
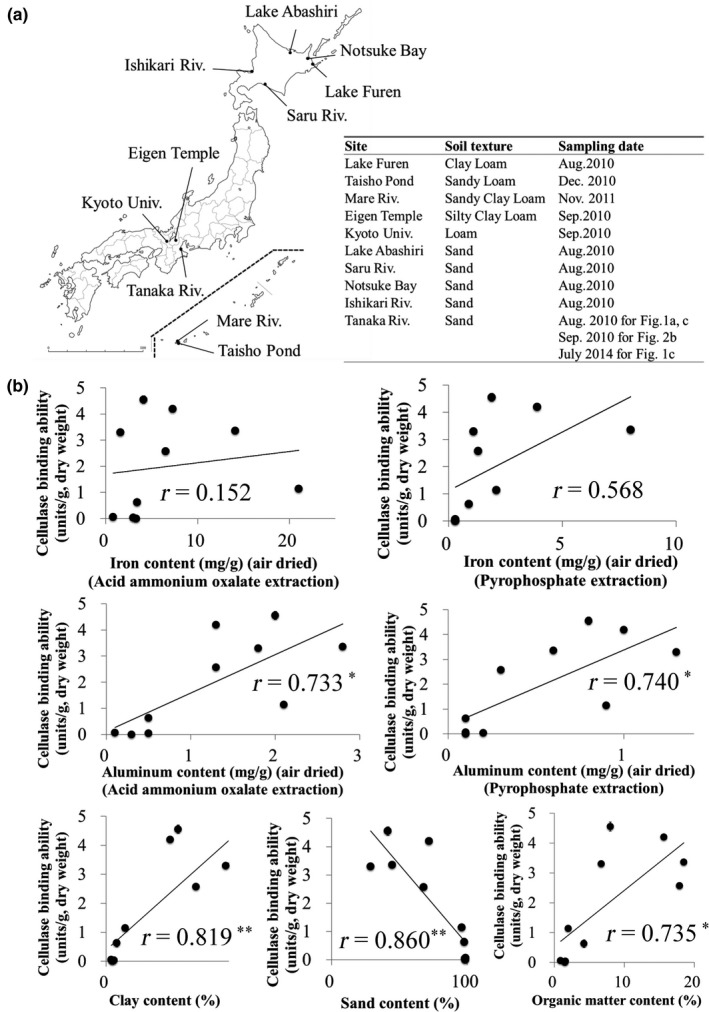
Correlations between the *Corbicula japonica*‐cellulase‐binding ability and soil textures of various sediments of Japan. (a) Sediment used for investigating the correlations between the *C. japonica*‐cellulase‐binding abilities and soil textures. The locations of sampling sites are shown on the map of Japan. The soil texture and sampling date of all sites are shown in the table beside the map. (b) The contents of iron and aluminum were measured by oxalate extraction and pyrophosphate extraction. Values of all soil properties are from a single measurement. Values of cellulase binding abilities are mean ± *SEM* (*n* = 3). The correlations are shown as Pearson's correlation coefficient (*r*). One asterisk indicates significance at the 0.05 level, and two asterisks indicate significance at the 0.01 level, both 2‐tailed. The binding ability is positively related to the aluminum content, the clay content, and the organic matter content, and negatively related to the sand content

In a previous study, we discovered that the environmental cellulase activities were highly correlated to the content of organic matter in the sediment, which in that case was mostly plant residues. In the present study, we separated the visible plant residues from the sediment and measured the cellulase activities of both the plant residues and the remaining mineral fraction. The results showed that the cellulase activity in the sediment originated mainly from the plant residues, but also partially from the mineral fraction (Figure 1d). Also, we investigated the correlation coefficient between the *C. japonica*‐cellulase‐binding ability and the various properties (iron content, aluminum content, sand content, clay content, and organic matter content) of the sediments collected from various locations in Japan. The results showed that the cellulase‐binding ability of sediments was positively related to the aluminum content (*r* = 0.733, *p* < 0.05 and *r* = 0.740, *p* < 0.05, respectively for acid ammonium oxalate extraction and pyrophosphate extraction), the content of clay (*r* = 0.819, *p* < 0.01), and the organic matter content (*r* = 0.735, *p* < 0.05), and negatively related to the sand content (*r* = 0.860, *p* < 0.01) (Figure 2).

### 3.3 Reconstruction experiment to reproduce the secretion and immobilization of environmental cellulase

**Figure 3:**
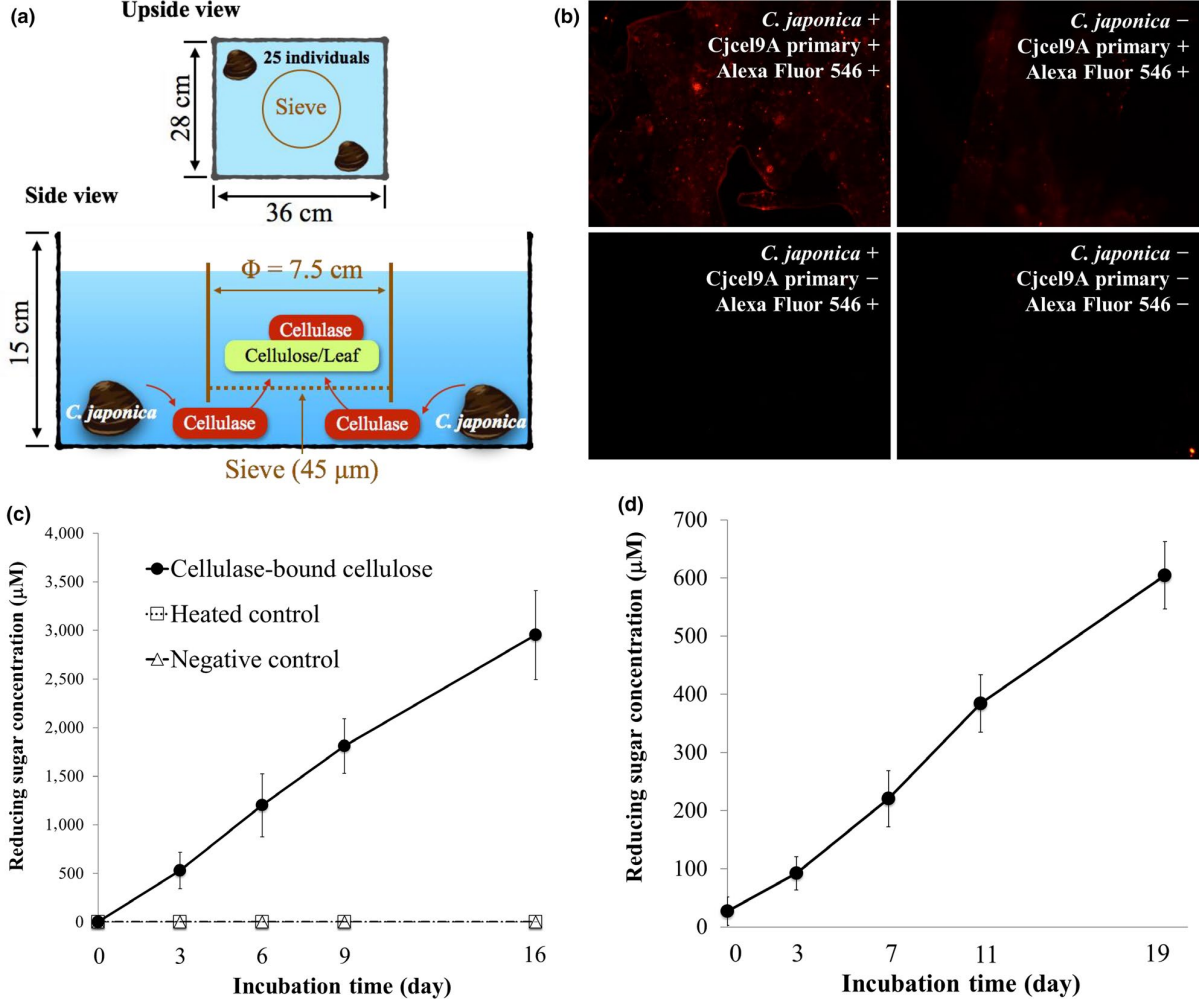
Reconstruction experiment. (a) Scheme of the reconstruction experiment. Twenty‐five *Corbicula japonica* individuals were cultured outside a 45‐µm sieve, which contained autoclaved plant residue (reed leaves) or pure cellulose, in a culture tank 36 cm in length, 28 cm in width, and 15 cm in depth. After a 24‐hr culture, the plant residue or cellulose was collected for immunological detection or activity assays. (b) Immunofluorescent staining of Cjcel9A bound to cellulose collected in the reconstruction experiment. Primary antibody dilution ratio, 1:1,000. Secondary antibody dilution ratio, 1:500. Scale bar, 100 µm, applicable in all images. Cjcel9A was detected on the cellulose in the presence of *C. japonica*. (c) Reducing sugar production from the cellulose collected in the reconstruction experiment. The concentration of reducing sugar increased over time in the presence of antimicrobial agent (10 mM NaN_3_), indicating the existence of bound cellulase. Values are mean ± *SEM* (*n* = 5). (d) Reducing sugar production from the plant residues collected in the reconstruction experiment. The concentration of reducing sugar increased over time in the presence of antimicrobial agent (10 mM NaN_3_), indicating the existence of bound cellulase. Values are mean ± *SEM* (*n* = 3)

To verify the secretion of *C. japonica* cellulase, a reconstruction experiment was conducted, as shown in Figure 3a. As a result, Cjcel9A was immunologically detected in the separated cellulose collected after a 1‐day culture (Figure 3b). In addition, the apparent size of Cjcel9A was identical to the one detected in the sediment collected in TRE (Figure 1c, Lane C and S, 60 kDa). Moreover, reducing sugar (products of the cellulose decomposition) was detected from either the pure cellulose or the leaves (natural cellulose origin) that were collected from the reconstruction experiment and incubated separately afterward (Figure 3c,d). As shown in Table 1, significantly more reducing sugar was produced from cellulase‐bound cellulose than from the heated control or negative control from Day 3 until Day 16 of the incubation. Within the group of cellulase‐bound cellulose, significantly more production of reducing sugar appeared from Day 6 compared to the start of the incubation (Day 0). As shown in Table 2, within the group of cellulase‐bound plant residue (reed leaves), significant production of reducing sugar appeared from Day 11, which was later than that for cellulase‐bound cellulose. As shown in Table 3, significantly more glucose was produced from cellulase‐bound cellulose than from the heated control or negative control from Day 3 until Day 16. Within the group of cellulase‐bound cellulose, a significant difference of glucose production appeared from Day 16, which was later than that of reducing sugar production (Table 1).

**Table 1:** Significance of the reducing sugar production from the cellulose that was collected in the reconstruction experiment

Significance	Reducing sugar concentration (cellulase‐bound cellulose)			
	3 days	6 days	9 days	16 days
Reducing sugar concentration (heated control)				
3 days	<0.001**			
6 days		0.001**		
9 days			<0.001**	
16 days				<0.001**
Reducing sugar concentration (negative control)				
3 days	<0.001**			
6 days		<0.001**		
9 days			<0.001**	
16 days				<0.001**
Reducing sugar concentration (cellulase‐bound cellulose)				
0 day	0.440	0.034*	0.002*	<0.001**

Note

One asterisk indicates *p* ≦ 0.05, while two asterisks indicate *p* ≦ 0.001.

**Table 2:** Significance of the reducing sugar production from the plant residue (reed leaves) that was collected in the reconstruction experiment

Significance	Reducing sugar concentration (plant residue)			
	3 days	7 days	11 days	19 days
Reducing sugar concentration (plant residue)				
0 day	0.825	0.065	0.001**	<0.001**

Note

One asterisk indicates *p* ≦ 0.05, while two asterisks indicate *p* ≦ 0.001.

**Table 3:** Significance of the glucose production from the cellulose that was collected in the reconstruction experiment

Significance	Glucose concentration (Cellulase‐bound cellulose)			
	3 days	6 days	9 days	16 days
Glucose concentration (heated control)				
3 days	0.012**			
6 days		<0.001**		
9 days			0.002*	
16 days				0.006*
Glucose concentration (negative control)				
3 days	0.009*			
6 days		<0.001**		
9 days			0.001**	
16 days				0.005*
Glucose concentration (cellulase‐bound cellulose)				
0 day	0.560	0.440	0.284	0.005*

Note

One asterisk indicates *p* ≦ 0.05, while two asterisks indicate *p* ≦ 0.001.

### 3.4 Effect of various saccharides on *C. japonica* cellulase secretion

**Figure 4:**
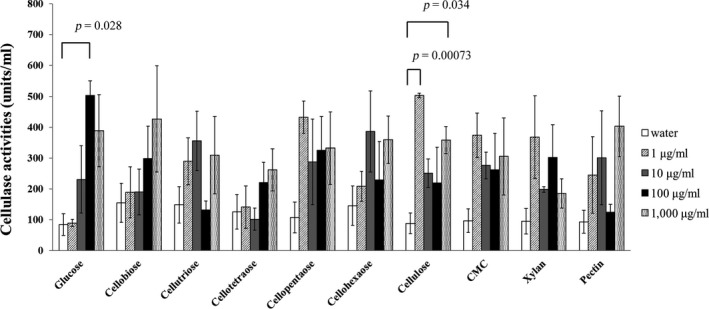
Effect of added saccharides on *Corbicula japonica* cellulase secretion. Ten kinds of poly‐, oligo‐, and monosaccharides at four concentrations (1, 10, 100, and 1,000 µg/ml) were investigated for their ability to affect the secretion of *C. japonica* cellulase. Glucose (100 µg/ml) and cellulose (1 µg/ml, 1,000 µg/ml) significantly triggered cellulase secretion. The significance was shown as *p* value. Values are mean ± *SEM* (*n* = 3)

As shown in Figure 4, among several polysaccharides (substrates of cellulase), oligosaccharides, and monosaccharides (both products of cellulase) tested, we found that cellulose at 1 µg/ml could significantly increase the cellulase secretion of *C. japonica*. Cellulose at 1,000 µg/ml could also increase the secretion, but the effect was weaker than that at 1 µg/ml, while no significant effect was found at 10 or 100 µg/ml. On the other hand, glucose, which is the final product of cellulose decomposition, increased cellulase secretion most strongly at 100 µg/ml. The effect seemed to increase from low concentration to high, and to be maximal at 100 µg/ml, but no significant difference was detected among 1, 10, or 1,000 µg/ml of glucose. As for the oligosaccharides, no significant effects on cellulase secretion were detected at any concentration. The same result was found using carboxymethyl cellulose (CMC), which is normally used in assays for the detection of cellulase activity because of its solubility. Also, no significant effects were found for xylan or pectin, known as hemicellulose, which are also components of the plant cell wall.

### 3.5 Glucose uptake

**Figure 5:**
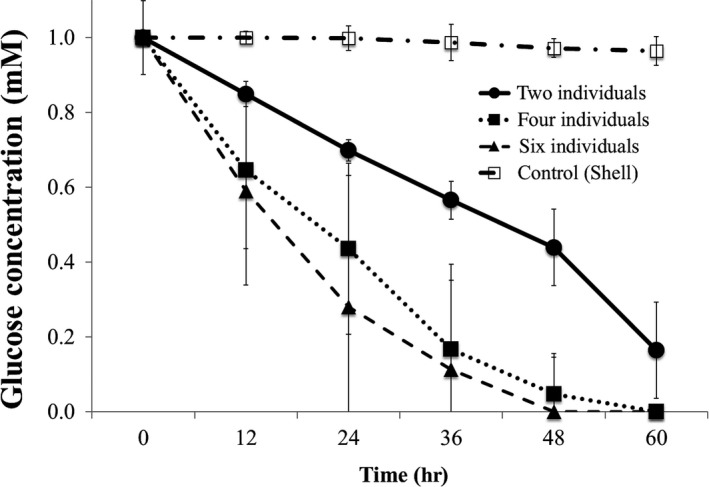
Glucose uptake by *Corbicula japonica* individuals. The concentration of glucose in the culture solution continually decreased as a function of culture time, or of the number of *C. japonica* individuals. Values are mean ± *SEM* (*n* = 3)

Glucose is the final product of cellulose decomposition. We investigated whether *C. japonica* is capable of taking up glucose produced in seawater. The results showed that the concentration of glucose decreased as a function of the culture time, and the rate of decrease was related to the number of *C. japonica* individuals (Figure 5). As shown in Table 4, compared to the glucose concentration in the control culture (*C. japonica* shells), the glucose concentration decreased significantly more quickly from 48 hr onwards after incubation started in the presence of 2 *C. japonica* individuals. A significant increase of glucose loss was detected earlier when the number of *C. japonica* individuals was increased to 4 or 6 (starting at 36 hr and 24 hr after the incubation started in the presence of 4 and 6 individuals, respectively). Within each group, significant differences appeared starting at 12 hr after incubation began in the presence of 2 individuals, while they appeared later in the presence of 4 and 6 individuals (starting at 48 hr in the presence of 4 individuals, and starting at 60 hr in the presence of 6 individuals).

**Table 4:** Significance of the glucose production from the cellulose that was collected in the reconstruction experiment

Significance	Glucose concentration (two individuals)					Glucose concentration (four individuals)					Glucose concentration (six individuals)				
	12 hr	24 hr	36 hr	48 hr	60 hr	12 hr	24 hr	36 hr	48 hr	60 hr	12 hr	24 hr	36 hr	48 hr	60 hr
Glucose concentration (control)															
12 hr	0.619					0.098					0.055				
24 hr		0.500					0.096					0.033*			
36 hr			0.181					0.009*					0.006*		
48 hr				0.014*					<0.001**					<0.001**	
60 hr					<0.001**					<0.001**					<0.001**
Glucose concentration (two individuals)															
0 hr	0.024*	0.001**	<0.001**	<0.001**	<0.001**										
Glucose concentration (four individuals)															
0 hr						0.981	0.711	0.075	0.008*	<0.001**					
Glucose concentration (six individuals)															
0 hr											1.000	0.898	0.501	0.064	0.003*

Note

One asterisk indicates *p* ≦ 0.05, while two asterisks indicate *p* ≦ 0.001.

### 3.6 Contribution of *C. japonica* to the cellulase activity in wetland sediments

We estimated the efficiency of cellulase secretion (ECS) to be 253.5–1509.1 units per *C. japonica* individual. In the sediment of Sandy field of TRE, we estimated the number of *C. japonica* to be approximately 50 per 1 m^3^. Also, we calculated the average cellulase activity as 0.122 unit per cm^3^. Thus, we finally estimated the contribution of *C. japonica* to the total cellulase activity to be approximately 10.4%–61.8% in the sediment of Sandy field of TRE. This estimate would be expected to be greatly influenced by the level of cellulase secretion as well as the number of *C. japonica*.

## 4 DISCUSSION

In the present study, we unexpectedly discovered the existence of a cellulase of *C. japonica*, namely Cjcel9A, in the sediment of a wetland. To reveal which part of the sediment (minerals or organic matter) immobilizes the cellulase, we separated the visible plant residues (the main organic matter at Sandy field in TRE, since the ignition loss rates were almost the same with or without removal of the plant residues) from the sediment and measured the cellulase activities associated with both the plant residues and the remaining minerals. The results showed that the cellulase activity in the sediment originated mainly from the plant residues (Figure 1d). This fact was verified by investigating the correlation coefficient between the *C. japonica*‐cellulase‐binding ability and the various properties of the sediments collected in Japan, which revealed that the binding ability is proportional to the organic matter content (Figure 2). These facts suggested that plant residues might be responsible for the fact that secreted cellulase is not washed away. Moreover, because cellulose is the substrate for cellulases, it is not surprising that the cellulase could bind to the cellulose contained in the plant residues. Cellulose contained in plant residues in the wetland sediment, in this case, acts as a scaffold to immobilize cellulases secreted by *C. japonica*.

In the controlled laboratory environment (Figure 3a), autolytic cellulase activity was detected in the plant residues (reed leaves collected in TRE) in the system, suggesting that cellulases of *C. japonica* were secreted and bound to the plant residues, since the plant residues were autoclaved prior to the experiment to eliminate all enzymatic activities. However, we still cannot exclude the possibility that this activity originated from some microbial population with cellulase activity. This is because we could not kill all microbes, such as those inside *C. japonica* individuals. By substituting plant residues for pure cellulose, we succeeded in immunological detection of Cjcel9A (Figure 1c Lane C and Figure 3b), which clearly demonstrated two facts: (a) *C. japonica* secreted Cjcel9A into the environment and (b) secreted Cjcel9A was immobilized on cellulose in the environment. In addition, the molecular size of the Cjcel9A detected in the reconstruction experiment was the same as that in wetland sediment, suggesting that this system is actually operating in natural conditions. On the other hand, the *C. japonica*‐cellulase‐binding ability was also positively related to the aluminum content of the sediment (Figure 2b). This relationship, however, is considered to be indirect because organic matter in sediments such as plant residues has a strong positive relationship to the content of aluminum oxides (Mikutta, Kleber, & Jahn, 2005; Watanabe, 2017).

On the other hand, although cellulase that was bound to either cellulose or plant residue could produce reducing sugar (Figure 3c,d), this production occurred faster on cellulose than on plant residue (Tables 2 and 3). This result suggests that pure cellulose interacts more efficiently with the enzyme, possibly because the plant residues used in this experiment expose less cellulose for cellulase attack, or because cellulose was already partially decomposed when the plant residues were collected.

**Figure 6:**
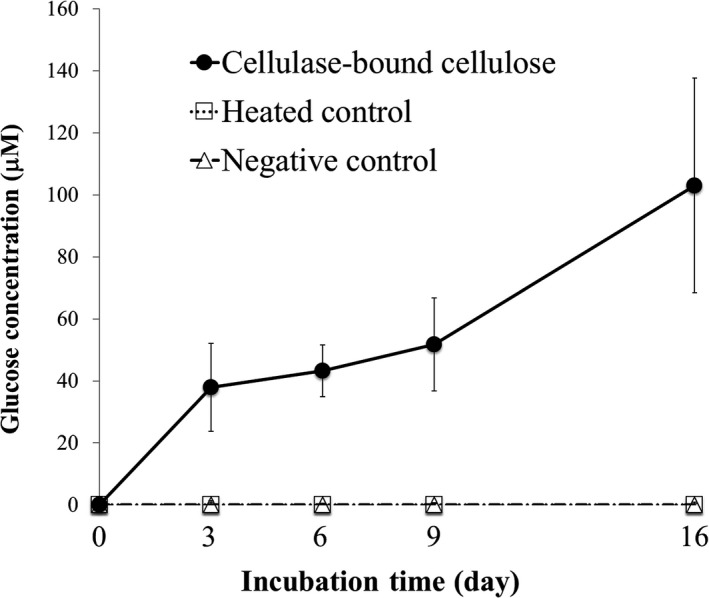
Glucose production from the cellulose collected in the reconstruction experiment. The concentration of glucose increased over time in the presence of antimicrobial agent (10 mM NaN_3_), indicating the existence of bound β‐glucosidase, which cleaves oligosaccharides into glucose. Values are mean ± *SEM* (*n* = 5)

Interestingly, although there are multiple forms of Cjcel9A in the enzyme storage organ called the “crystalline style” and the digestive gland mixture of *C. japonica* (Figure 1c Lanes CS and DG), only one form of Cjcel9A (60 kDa) was secreted (Figure 1c Lanes S and C). The cellulase corresponding to the major form (67 kDa) might be a precursor of Cjcel9A. Moreover, glucose was produced from cellulose added to cultures of *C. japonica* (Figure 6). Table 3 shows that significant production of glucose appeared later than that of reducing sugar (Table 1), which is reasonable because glucose production requires the production of oligosaccharides first. Since Cjcel9A is considered to be an endo‐β‐glucanase, which can only cleave cellulose into oligosaccharides, the production of glucose thus indicates the existence of β‐glucosidase simultaneously secreted by *C. japonica*. Although endogenous β‐glucosidase of *C. japonica* was previously reported (Sakamoto, Uji, Kurokawa, & Toyohara, 2009), immunological detection will be needed to examine its secretion.

Because the antibody raised against Cjcel9A is thought to only identify the cellulase of *C. japonica*, we thus concluded that at least a part of the environmental cellulase is secreted by *C. japonica*, but the question of whether it is physiologically secreted or not remains. If it is physiologically secreted, the secretion could be regulated by its substrate, namely cellulose. Moreover, Figure 3c,d and Figure 6 show that the secreted cellulase could produce oligosaccharides and glucose. These products could also affect the secretion level. As shown in Figure 4, cellulase secretion increased most markedly in the presence of glucose, especially at the concentration of 100 µg/ml. The effect of cellulose was higher at cellulose concentration of 1 µg/ml than at 1,000 µg/ml. This might be because the amount of cellulase secreted from *C. japonica* was maximally induced in the presence of 1 µg/ml cellulose, and the level of glucose produced would not increase in the presence of a higher concentration of cellulose.

In contrast, oligosaccharides had no effect on the cellulase secretion, possibly because cellulases secreted from *C. japonica* must rely on long cellulose chains rather than short oligosaccharides for forming a complex necessary for the effective production of glucose. In addition, CMC showed no significant effect, possibly because the carboxymethyl groups interfered with its recognition by *C. japonica*. Moreover, hemi‐cellulose of xylan and pectin also had no effect. Since previous studies have shown the existence of xylose and pectinase in *C. japonica* (Sakamoto & Toyohara, 2009), the present finding suggests that their products have no effect on cellulase secretion. Those findings suggest that *C. japonica* might use glucose sensor(s) to monitor the environmental cellulose in order to control the level of its cellulase secretion. However, proving this conjecture would need further evidence, such as revealing the molecular mechanism of the cellulase expression/secretion. In addition, besides being possibly used as a signal for the existence of environmental cellulose, glucose was efficiently taken up by *C. japonica*, possibly as a nutritional source (Figure 5). We showed that the more *C. japonica* individuals were present, the more quickly they absorbed the glucose in the culture water (Table 4). However, Table 4 also suggests that there might be big differences among *C. japonica* individuals in the behavior of glucose uptake, because within each group the significant effects appeared later (with greater variation) when the number of individuals increased.

Recent molecular biological studies suggest that a variety of aquatic invertebrates from a wide range of phyla possess endogenous cellulase (Guo, Ding, Zhang, Xu, & Zhao, 2008; Nishida et al., 2007; Suzuki, Ojima, & Nishida, 2003). Our previous studies suggested that in some low‐temperature wetlands in Hokkaido, Japan, small‐sized meiobenthos could also be involved in cellulose breakdown (Toyohara, Park, Tsuchiya, & Liu, 2012). Another previous study of ours showed that planktonic crustaceans in tropical areas, such as the mangrove forest in Malaysia, consume land‐originating cellulose directly, since the photosynthetic microalgae were insufficient there due to the low transparency of the water (Liu et al., 2015). Invertebrates have thus increasingly been shown to be important in the process of wetland cellulose decomposition. Nevertheless, few previous studies attempted to reveal the molecular mechanism of this process.

**Figure 7:**
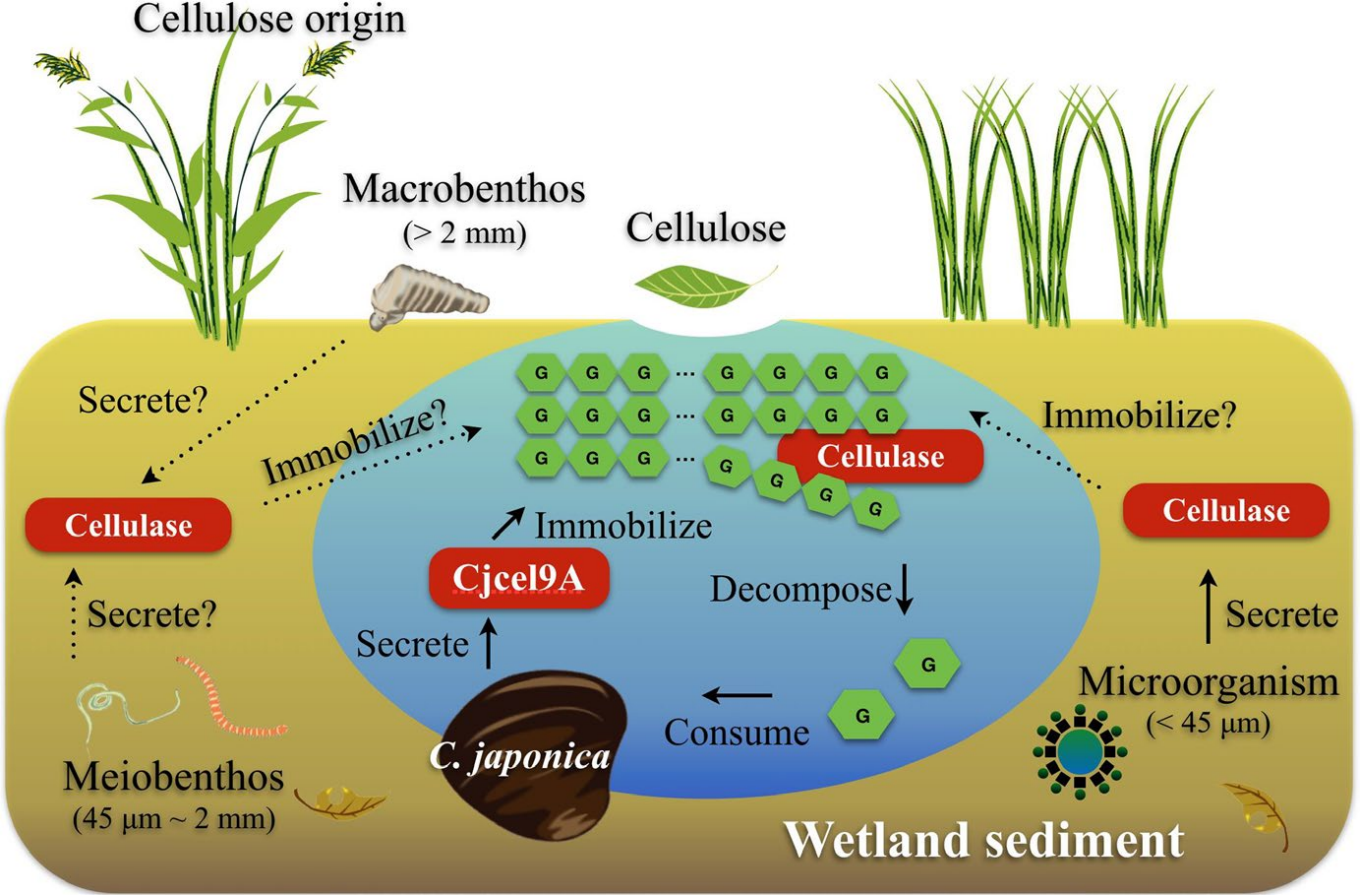
Scheme of the “environmental bioreactor system” in wetland. Solid lines are used to show the findings in previous studies or the present study. Dotted lines are used to show the prediction that is based on our findings. *Corbicula japonica* exemplifies the bivalves that secrete cellulase into the environment. Secreted cellulase forms an immobilized complex with environmental cellulose (e.g., leaves). Cellulose is continually being decomposed into oligosaccharides and glucose by immobilized cellulase. Glucose is consumed by *C. japonica*, and it has the effect of increasing the cellulase secretion of *C. japonica*. As for other organisms, microorganisms are already known to secrete cellulases, and thus their cellulases could also be immobilized on environmental cellulose. This phenomenon might also be seen for other macrobenthos and meiobenthos, but requires further investigations. If the cellulase secretion is universal, this system could be a key component of the cellulose decomposition in wetlands

The present study revealed a novel decomposition system, which we call an “environmental bioreactor system” (Figure 7). *Corbicula japonica* is the first invertebrate model organism shown to secrete endogenous cellulase into the environment. The estimated contribution of *C. japonica* to environmental cellulase is approximately 10.4%–61.8% in TRE. Although this percentage is greatly influenced by the number of as well as the cellulase secretion level of *C. japonica* individuals, the contribution of *C. japonica*'s secreted cellulase to the environmental bioreactor system should not be ignored, at least in this habitat. Questions such as “how do cellulase providers like *C. japonica* maximize their benefits (because glucose is a universal energy source for all living creatures, and thus could be easily consumed when generated in the environment)?” and “is this phenomenon restricted to *C. japonica*, or to bivalves, or is it in common use by other aquatic invertebrates and microorganisms?” are yet to be further investigated and answered.

If cellulase secretion is common, an environmental bioreactor system could be a key function in all wetlands. In the system studied here, the secreted cellulase, which intrinsically enables various biological activities inside its providers, was immobilized by the sediment, becoming a part of the common environmental materials after it was secreted. Wetlands are thus like scaffolds that immobilize secreted cellulase, allowing themselves to become a kind of “breakdown factory” that benefits the whole area, even when the enzyme providers are resting or absent. To confirm this, however, investigations of matters such as the environmental cellulase activities in other types of wetlands, or immunological detection to test for the existence of other invertebrates’ cellulases in wetland sediments, will be required.

## CONFLICT OF INTEREST

We declare that we have no significant competing financial, professional, or personal interests that might have influenced the performance or presentation of the work described in this manuscript.

## AUTHOR CONTRIBUTIONS

W. L. designed the experiments, analyzed results, and wrote the paper. A. T. and Y. N. performed sampling, designed the biochemical experiments, and analyzed results. T. W. advised about the soil property analysis. S. M. and H. T. supervised and advised throughout the experiments and the paper writing.

## Data Availability

All quantitative data (environmental cellulase activities data in Figure 1, soil texture data in Figure 2, reconstruction experiment related data in Figures 3 and 6, saccharides effect related data in Figure 4, and glucose uptake data in Figure 5) could be downloaded from Dryad (https://doi.org/10.5061/dryad.8mg5702). DNA sequence of Cjcel9A: GenBank: AB264777.1.
